# Functional role of IL-22 in psoriatic arthritis

**DOI:** 10.1186/ar3781

**Published:** 2012-03-14

**Authors:** Anupam Mitra, Smriti K Raychaudhuri, Siba P Raychaudhuri

**Affiliations:** 1 University of California, Davis, School of Medicine, Dermatology, Davis & VA Medical Center Sacramento, Mather, CA, USA; 2 University of California, Davis, School of Medicine, Medicine/Rheumatology, Allergy & Clinical Immunology, Davis & VA Medical Center Sacramento, Mather, CA, USA

## Abstract

**Introduction:**

Interleukin-22 (IL-22) is a cytokine of IL-10 family with significant proliferative effect on different cell lines. Immunopathological role of IL-22 has been studied in rheumatoid arthritis (RA) and psoriasis. Here we are reporting the functional role of IL-22 in the inflammatory and proliferative cascades of psoriatic arthritis (PsA).

**Method:**

From peripheral blood and synovial fluid (SF) of PsA (*n *= 15), RA (*n *= 15) and osteoarthritis (OA, *n *= 15) patients, mononuclear cells were obtained and magnetically sorted for CD3^+ ^T cells. Fibroblast like synoviocytes (FLS) were isolated from the synovial tissue of PsA (*n *= 5), RA (*n *= 5) and OA (*n *= 5) patients. IL-22 levels in SF and serum were measured by enzyme linked immunosorbent assay (ELISA). Proliferative effect of human recombinant IL-22 (rIL-22) on FLS was assessed by MTT (3-(4, 5-Dimethylthiazol-2-yl)-2, 5-diphenyltetrazolium bromide, a yellow tetrazole) and CFSE dilution (Carboxyfluorescein succinimidyl ester) assays. Expression of IL-22Rα1 in FLS was determined by western blot.

**Results:**

IL-22 levels were significantly elevated in SF of PsA patients (17.75 ± 3.46 pg/ml) compared to SF of OA (5.03 ± 0.39 pg/ml), p < 0.001. In MTT and CFSE dilution assays, rIL-22 (MTT, OD: 1.27 ± 0.06) induced significant proliferation of FLS derived from PsA patients compared to media (OD: 0.53 ± 0.02), p < 0.001. In addition, rIL-22 induced significantly more proliferation of FLS in presence of TNF-α. IL-22Rα1 was expressed in FLS of PsA, RA and OA patients. Anti IL-22R antibody significantly inhibited the proliferative effect of rIL-22. Further we demonstrated that activated synovial T cells of PsA and RA patients produced significantly more IL-22 than those of OA patients.

**Conclusion:**

SF of PsA patients have higher concentration of IL-22 and rIL-22 induced marked proliferation of PsA derived FLS. Moreover combination of rIL-22 and TNF-α showed significantly more proliferative effect on FLS. IL-22Rα1 was expressed in FLS. Successful inhibition of IL-22 induced FLS proliferation by anti IL-22R antibody suggests that blocking of IL-22/IL-22R interaction may be considered as a novel therapeutic target for PsA.

## Introduction

Psoriatic arthritis (PsA) is an inflammatory arthritis associated with psoriasis [1,2]. The role of genetic, immunological and environmental factors in the pathogenesis of PsA has been proposed, however the exact cause remains to be determined [3,4]. In the joint of PsA patients, there is hyperplasia of synovial lining cells and infiltration of mononuclear cells, which plays an important role in disease pathogenesis [5]. In PsA activated T cells produce increased amount of pro-inflammatory cytokines like IL-1β, IL-2, IFN-γ, TNF-α and IL-17 in the synovial fluid (SF) and thus contribute significantly to the disease pathogenesis [6-8]. Moreover fibroblasts isolated from skin and joints of patients with PsA showed increased proliferative activity and are capable of secreting IL-6, IL-1 and platelet derived growth factor [9]. It was reported previously that Th1 cytokines like IL-1β, TNF-α were higher in PsA compared to rheumatoid arthritis (RA) [6]. Recent findings suggest that there is hardly any difference in Th1 cytokines among these two groups [10]. The pro-inflammatory cytokine, TNF-α stimulates the proliferation of synovial fibroblasts leading to formation of pannus [11,12], as well as induces expression of chemokines and adhesion molecules which play important role in the pathogenesis of RA [13]. In most of the RA and PsA patients, neutralization of pro-inflammatory cytokines improve the disease condition but there are some patients who do not respond to this therapy [14,15]. This indicates that there are other cytokines and growth factors, which may play important roles in the disease process.

IL-22, a newly discovered Th17 cytokine belongs to cytokine of IL-10 family, however differs from other cytokines of IL-10 family by being a potent proliferative and inflammatory agent for different cell lines [16-20]. Activated T cells of Th17 and Th22 subsets are the major source of IL-22 [21-24]. Apart from activated T cells, IL-22 can be produced by natural killer (NK) cells also [25]. IL-22 acts through IL-22 receptor, which is a complex of IL-22R1 and IL-10R2 [26]. IL-10R2 is ubiquitously expressed whereas IL-22R1 is expressed in liver, colon, small intestine, pancreas, kidney, skin and fibroblast like synoviocytes of joints [20,27]. In the pathogenesis of various autoimmune diseases such as psoriasis and RA, the role of IL-22 has been established [18-20,28]. Moreover, in collagen induced arthritis animal model, IL-22 has been found to play an important role in pannus formation as well as in osteoclastogenesis [29].

There is only one study which reported elevated levels of IL-22 in synovial fluid (SF) of PsA patients [30]. To our knowledge, there is no reported study on functional role of IL-22 in PsA. In this study we investigated the functional role of IL-22 in PsA. We measured IL-22 levels in serum and synovial fluid (SF) of PsA, RA and OA. To determine the functional significance, we evaluated the proliferative effect of human recombinant IL-22 (rIL-22) on fibroblast like synoviocytes (FLS) isolated from PsA synovial tissue. Expression of functional receptor of IL-22, IL-22Rα1 was determined at protein level in FLS. Further we examined the inhibitory effect of anti IL-22R antibody on IL-22 induced FLS proliferation in PsA.

## Materials and methods

### Study population

This study was approved by the Institutional Review Board (IRB) of Veterans Affairs Medical Centre (VAMC), Sacramento. After obtaining IRB approved informed consent form, specimens were collected from patients with active PsA, RA and OA. RA and OA patients were recruited according to the clinical, laboratory and radiographic classification criteria of the American College of Rheumatology (ACR). Patients with PsA had either oligoarthritis or symmetric polyarthritis as described by Moll and Wright [31]. All patients with PsA fulfilled the CASPAR classification criteria for PsA [32]. Before enrolling in this study, patients had undergone complete physical examination, evaluation of severity of psoriasis and arthritis, appropriate blood tests and radiological studies. Active PsA or RA was defined by the presence of at least 3 swollen and 3 tender joints. In patients with PsA, in addition to the above, presence of plaque psoriasis with a qualifying lesion of at least 2 cm in diameter was checked. All patients were evaluated for the swollen joint count, the tender joint count, patient's assessment of pain, ESR and CRP.

SF and blood were collected from patients with PsA (*n *= 15), RA (*n *= 15) and OA (*n *= 15). Synovial fluid mononuclear cells (SFMC) and peripheral blood mononuclear cells (PBMC) were isolated from SF and blood respectively. SF and synovial tissues were collected from one or both knees of PsA, RA and OA patients. Synovial tissues were studied from PsA (*n *= 5), RA (*n *= 5) and OA (*n *= 5) patients. These subjects had an arthroscopic procedure or joint replacement for treatment of their arthritis. All enrolled patients were on NSAID or Acetaminophen except two RA patients were on low dose oral steroid (≤ 7.5 mg/day), six RA patients were on hydroxychloroquine; six RA and five PsA patients were on low dose methotrexate (< 10 mg/week). None of the PsA patients was on steroid. None of the RA or PsA patients was on biologics for at least 3 months before the enrollment for this study.

### ELISA of IL-22 in synovial fluid (SF) and serum of patients with PsA, RA and OA

Peripheral blood and SF from the knee joints of patients with PsA (*n *= 15), RA (*n *= 15) and OA (*n *= 15) were collected. The SF was immediately centrifuged at 1600 rpm for 20 min at 4°C to remove cells and debris. SF supernatants were carefully removed and stored at -20°C for ELISA of IL-22 (Human IL-22 ELISA Kit, eBioscience, San Diego, CA, USA). Serum was stored at -20°C for ELISA of IL-22. Sensitivity for IL-22 detection by this kit is 8-1000 pg/ml.

### Functional role of human rIL-22 in FLS of patients with PsA

#### 1. Isolation of FLS from synovial tissue

Fibroblast like synoviocytes (FLS) were isolated from synovial tissue biopsies of PsA (*n *= 5), RA (*n *= 5) and OA (*n *= 5) according to our standardized protocol [33,34]. FLS were cultured in DMEM (Mediatech, Manassas, VA, USA), supplemented with sodium pyruvate (Mediatech, Manassas, VA, USA), penicillin and streptomycin (Axenia BioLogix, Dixon, CA, USA) and 10% (v/v) fetal bovine serum (Axenia BioLogix, Dixon, CA, USA) (complete DMEM) at 37°C in a humidified atmosphere of 95% air and 5% CO2. After the third passage, all FLS were examined for the fibroblast specific markers ASO2 (EMD4Biosciences, San Diego, CA, USA) and CD55 (BD Pharmingen, San Diego, CA, USA). No expression of the macrophage specific marker CD68 (BD Pharmingen, San Diego, CA, USA) or pan endothelial cell adhesion molecule (PECAM), CD31 (BD Pharmingen, San Diego, CA, USA) were detected in FLS cultures, thus excluding possible contamination of synovial fibroblasts by synovial macrophages and endothelial cells. All experiments were performed with cells between 3^rd ^and 6^th ^passages.

#### 2. Proliferative effect of human rIL-22 in FLS of PsA, RA and OA patients

The proliferative effect of human rIL-22 (eBioscience, San Diego, CA, USA) in FLS of PsA, RA and OA patients was assessed *in vitro *by MTT assay and Carboxy fluorescein succinimidyl ester (CFSE) dilution assay.

a. MTT assay- FLS from PsA (*n *= 5), RA (*n *= 5) and OA (*n *= 5) were used to determine the IL-22 induced proliferative effect. Human recombinant TNF-α (rTNF-α) was used as a positive control for proliferation assays [11,12]. We performed dose response curve and observed that human rIL-22 at 100 ng/ml, human anti IL-22R antibody (IL-22R Ab., R&D Systems, Minneapolis, MN, USA) at 1000 ng/ml and human rTNF-α (eBioscience, San Diego, CA, USA) at 20 ng/ml showed optimum response. We used these concentrations in subsequent experiments. Third to sixth passage FLS (20,000 cells/well) were cultured in triplicate in 24 well plate for 5 days with medium only, rIL-22 (100 ng/ml), IL-22R Ab (1000 ng/ml), IL-22R Ab (1000 ng/ml) + rIL-22 (100 ng/ml), rTNF-α (20 ng/ml) and rIL-22 (100 ng/ml) + rTNF-α (20 ng/ml). MTT assay was done as per our standardized protocol [34]. Briefly, MTT (Sigma, St. Louis, MO, USA) 5 mg/ml was added to each well followed by incubation for 4 hours. Acid-isopropanol (0.04 N HCl in isopropanol, 100 μl/well) was added and plates were maintained at room temperature for 5 minutes. The optical density was measured using a microtiter plate reader at 570/690 nm.

b. Carboxy fluorescein succinimidyl ester (CFSE) dilution assay- We used Cell Trace CFSE cell proliferation kit (Molecular Probes, Eugene, OR, USA). For this assay, we seeded 2 × 10^5 ^FLS in T25 flask and stained them with CFSE (2 μM) followed by incubation with media only, rIL-22 (100 ng/ml), rTNF-α (20 ng/ml) and rIL-22 (100 ng/ml) + rTNF-α (20 ng/ml) for 5 days. CFSE dilution was evaluated by flow cytometry (FACScalibur, BD Biosciences, San Jose, CA, USA). Data were analyzed using FlowJo software (Treestar, Ashland, OR, USA).

### Western blot analysis of IL-22Rα1 expression in FLS of PsA, RA and OA

FLS of PsA, RA and OA were grown in T75 flask and upon 70-80% confluence, cells were scrapped with a cell scrapper, then centrifuged for 8 min at 1600 rpm, 22°C. Lysis buffer containing 150 mM NaCl (Fischer Scientific, Houston, TX, USA), 50 mM Tris-HCL of pH 7.2 (Sigma-Aldrich, St. Louis, MO, USA), 1% w/v Triton X-100 (Sigma-Aldrich, St. Louis, MO, USA), 1% w/v sodium deoxycholate (Sigma-Aldrich, MO, USA), 0.1% w/v SDS (Fischer Scientific, Houston, TX, USA) with protease inhibitor (Roche, Indianapolis, IN, USA) was added and incubated for 40 min at 4°C. Supernatants were collected after centrifugation at 4°C, 13000 rpm for 20 min and the protein concentration was estimated using BCA Protein Assay Reagent Kit (Thermo-Fisher scientific, Houston, TX, USA). Cell lysate protein (30 μg) was used for western blot analysis. Cell lysate was run in a 10% acrylamide gel (BIO-RAD, CA, USA). The Immuno-Blot PVDF membrane (BIO-RAD, Richmond, CA, USA) was blocked with 5% nonfat dried milk in TBST (50 ml of 1 M Tris, 30 ml of 3 M NaCl, 20 ml of 10% Tween, ddH2O, total volume 1L) for 2 hour at room temperature. Then blots were incubated overnight at 4°C with the primary antibody, IL-22Rα1 (R&D Sytems, Minneapolis, MN, USA) at 3 μg/ml concentrations; αtubulin (Cell signaling Technologies, Danvers, MA, USA), 1:250 concentrations. Blots were washed 5x in TBST and secondary antibody (Jackson Immunoresearch Labs, PA, USA) was added at 1:5000 dilution. After keeping the blot in secondary antibody for 1 hr, it was washed 5x in TBST and then developed using ECL western blotting detection reagents solution (Thermo-Fisher Scientific, Houston, TX, USA) for chemiluminescent detection. Band density was measured using Image J software (NIH, Bethesda, MD, USA).

### Inhibitory effect of human anti IL-22R antibody (IL-22R Ab) on rIL-22 induced FLS proliferation

FLS (20,000/well) were seeded in a 24-well plate with 1 ml of complete DMEM (DMEM, 10%FBS, antibiotics). Cells were pre-treated with IL-22R Ab (1000 ng/ml) for 1 hr at 37°C then human rIL-22 (100 ng/ml) was added and incubated at 37°C in CO2 incubator for 5 days. MTT assay was done to determine the inhibitory effect of IL-22R Ab on IL-22 induced FLS proliferation.

### Source of IL-22

To determine the source of IL-22, SFMC and PBMC isolated from SF and peripheral blood of PsA (*n *= 15), RA (*n *= 15) and OA (*n *= 15) were examined. PBMCs were isolated by Ficoll-hypaque gradient (GE Healthcare, Piscataway, NJ, USA) centrifugation of peripheral blood. Synovial fluid was treated with hyaluronidase enzyme (Sigma-Aldrich, MO, USA) at 1 mg/ml concentration for 5 mins at room temperature, then centrifuged at 2000 rpm for 20 min at 4°C. Cell pellet were resuspended in 1 ml of RPMI 1640 (Mediatech, Manassas, VA, USA) complete media (RPMI-1640 containing 10%FBS, antibiotics). Then the CD3^+ ^T cells were separated magnetically from SFMC and PBMC using Easy Sep negative selection human T cell enrichment kit (Stem Cell Technology, Vancouver, BC, Canada) according to manufacturer's protocol and the purity was determined by flow cytometry using human anti CD3 antibody (BD pharmingen, San Diego, CA, USA). In a 24-well tissue culture plate, 1 × 10^6 ^synovial CD3^+ ^T cells and peripheral blood CD3^+ ^T cells were seeded separately and stimulated with 50 ng/ml of phorbol myristate acetate (PMA) and 1 μg/ml of ionomycin (Sigma-Aldrich, MO, USA) for 24 hrs and 10 μg/ml of phytoheamagglutinin (PHA, Invitrogen, CA, USA) for 48 hrs. For mimicking physiological stimulation, FLS were cultured in 24-well plates coated with human anti CD3 and anti CD28 (5 μg/ml each) (BD Biosciences, San Diego, CA, USA) at 37°C. After 24 and 48 hrs, supernatant was collected and IL-22 was measured in the supernatant by ELISA.

### Statistical analysis

All experiments were done in triplicate. Results were expressed as Mean ± SEM. For analysis of flow cytometric data, we considered the % of divided cells of each treatment group using the FlowJo software (TreeStar Inc, Ashland, OR, USA). All data were analyzed statistically using the GraphPad Prism software version 5.0 (GraphPad Software Inc, La Jolla, CA, USA). Non-parametric tests (Wilcoxon matched pairs signed rank test, Mann Whitney test, Friedman's ANOVA, Kruskal Wallis ANOVA with Dunn's multiple comparison test) were used to determine the statistical significance. A p value < 0.05 was considered statistically significant.

## Results

### Synovial fluid IL-22 levels were elevated in PsA

**Figure 1:**
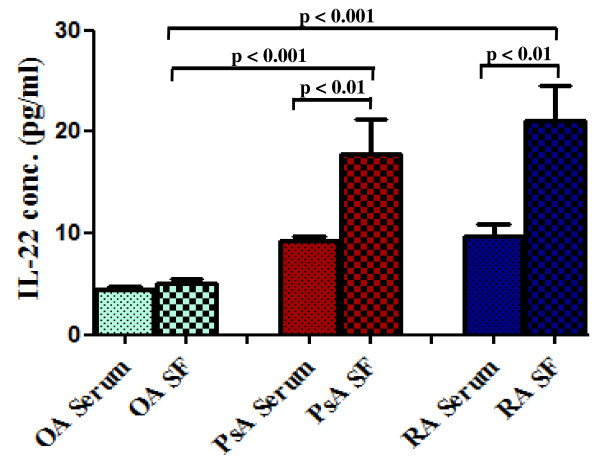
**IL-22 in SF and serum of PsA and RA were elevated compared to OA patients**. Synovial fluid (SF) and peripheral blood (PB) were collected from psoriatic arthritis (PsA, *n *= 15), rheumatoid arthritis (RA, *n *= 15) and osteoarthritis (OA, *n *= 15) patients. IL-22 levels were measured in SF and serum samples by ELISA. All experiments were done in triplicate. Results were expressed as Mean ± SEM. Nonparametric tests; Mann Whitney and Kruskal Wallis ANOVA with Dunn's multiple comparison tests were used to determine statistical significance.

IL-22 levels in SF of PsA (17.75 ± 3.46 pg/ml) were significantly higher compared to that in OA (5.03 ± 0.39 pg/ml, p < 0.001, Kruskal-Wallis ANOVA with Dunn's multiple comparison test) (Figure 1). In SF of RA, highest concentration of IL-22 (21.06 ± 3.55 pg/ml) was observed. However, there was no significant difference of IL-22 levels between SF of PsA (17.75 ± 3.46 pg/ml) and RA (21.06 ± 3.55 pg/ml). Serum IL-22 levels in PsA, RA and OA were 9.22 ± 0.54 pg/ml, 9.64 ± 1.20 pg/ml and 4.45 ± 0.25 pg/ml respectively, p = ns, Kruskal Wallis ANOVA with Dunn's multiple comparison test (Figure 1). We found significantly higher levels of IL-22 in SF of PsA (17.75 ± 3.46 pg/ml) and RA (21.06 ± 3.55 pg/ml) compared to that in serum of PsA (9.22 ± 0.54 pg/ml) and RA (9.64 ± 1.20 pg/ml) (p < 0.01, Mann Whitney test). In OA, there was no significant difference of IL-22 levels between SF (5.03 ± 0.39 pg/ml) and serum (4.45 ± 0.25 pg/ml).

### Human rIL-22 induced marked proliferation of FLS

**Figure 2:**
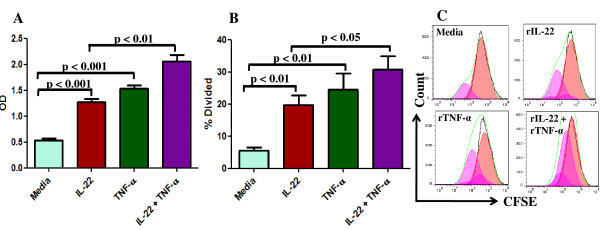
**Human rIL-22 induced marked proliferation of FLS in PsA patients**. FLS were isolated from synovial tissue of patients with psoriatic arthritis (PsA, *n *= 5). **(A) **FLS (20,000 cells/well) of PsA patients were seeded in a 24-well plate in triplicate and incubated for 5 days at 37°C in presence or absence of human rIL-22 (100 ng/ml), human rTNF-α (20 ng/ml) and combination of rIL-22 (100 ng/ml) + rTNF-α (20 ng/ml). FLS used in these experiments were between 3^rd ^and 6^th ^passage. MTT assay was done to determine FLS proliferation and results were expressed as Mean ± SEM of OD. (**B) **FLS (2 × 10^5 ^cells) of PsA patients were seeded in T25 flask in triplicate, stained with CFSE (2 μM) and incubated for 5 days at 37°C in presence or absence of human rIL-22 (100 ng/ml), human rTNF-α (20 ng/ml) and combination of rIL-22 (100 ng/ml) + rTNF-α (20 ng/ml). Data were acquired in FACScalibur flow cytometer and analysis was done using FlowJo software. Proliferation was measured in terms of percent of divided cells and data were expressed as Mean ± SEM. The bar diagram represents the percent divided cells of FLS in PsA patients. **(C) **A representative histogram showing the proliferative effect of different treatments (rIL-22, rTNF-α, rIL-22 + rTNF-α) compared to media in FLS obtained from PsA patients. Non parametric test, Friedman's ANOVA with Dunn's multiple comparison test was done to determine statistical significance.

MTT and CFSE dilution assay were done to determine the proliferative effect of human rIL-22 in FLS of PsA, RA and OA. In MTT assay, significantly higher proliferation of PsA derived FLS were observed with rIL-22 (OD: 1.27 ± 0.06) compared to media (OD: 0.53 ± 0.02, p < 0.001, Friedman's ANOVA with Dunn's multiple comparison test) (Figure 2A). In this experiment we used rTNF-α as a positive control [11,12] and significant proliferation were found with rTNF-α (OD: 1.53 ± 0.05) compared to media (OD: 0.53 ± 0.02, p < 0.001, Friedman's ANOVA with Dunn's multiple comparison test). There was no significant difference of proliferation between the rIL-22 (OD: 1.27 ± 0.06) and rTNF-α (OD: 1.53 ± 0.05) treated FLS. Here we observed a novel finding that rIL-22 induced significantly more proliferation in presence of rTNF-α (OD: 2.06 ± 0.11) than rIL-22 alone (OD: 1.27 ± 0.06, p < 0.01) (Figure 2A).

We also did this experiment in RA FLS and observed similar results; human rIL-22 (OD: 1.58 ± 0.05) and rTNF-α (OD: 1.96 ± 0.04) induced significant proliferation compared to media (OD: 0.65 ± 0.04, p < 0.001, Friedman's ANOVA with Dunn's multiple comparison test). In RA also, rIL-22 in presence of rTNF-α (OD: 2.37 ± 0.11) induced significantly more proliferation than rIL-22 alone (OD: 1.58 ± 0.05, p < 0.01). In OA FLS, rIL-22 (OD: 0.73 ± 0.12) as well rTNF-α (OD: 0.90 ± 0.16) induced significant proliferation compared to media (OD: 0.38 ± 0.05, p < 0.05, Friedman's ANOVA with Dunn's multiple comparison test).

We further confirmed the proliferative effect of human rIL-22 on FLS by CFSE dilution assay. In this assay, similar results were found regarding proliferation of FLS with rIL-22. In FLS of PsA, human rIL-22 (19.64 ± 3.13%) and human rTNF-α (24.60 ± 4.95%) induced significantly more proliferation compared to that in media only (5.58 ± 0.98%, p < 0.01, Friedman's ANOVA with Dunn's multiple comparison test) (Figure 2B and 2C). Here also, rTNF-α (24.60 ± 4.95%) showed more proliferation of FLS than rIL-22 (19.64 ± 3.13%), but there was no significant difference in proliferation of FLS between these two groups. In this assay also, rIL22 in presence of rTNF-α (30.76 ± 4.17%) showed more proliferation than rIL-22 alone (19.64 ± 3.13%, p < 0.05) (Figure 2B and 2C).

In RA patients also, rIL-22 (21.57 ± 3.33%) and rTNF-α (27.45 ± 3.29%) induced significant proliferation compared to that in media (6.43 ± 1.61%, p < 0.01, Friedman's ANOVA with Dunn's multiple comparison test). There was no significant difference of proliferation between rIL-22 and rTNF-α treated FLS. In RA also, we observed that rIL-22 induced significantly more proliferation of FLS in presence of rTNF-α (33.11 ± 2.42%) compared to rIL-22 alone (21.57 ± 3.33, p < 0.05).

### IL-22Rα1 was expressed in FLS

**Figure 3:**
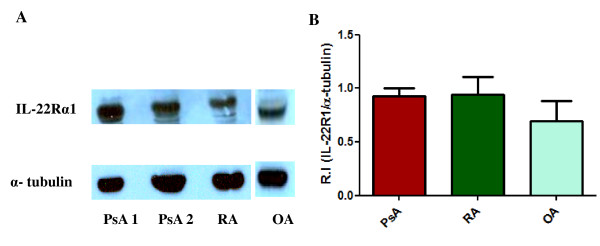
**Western blot analysis of IL-22Rα1 expression in FLS of PsA, RA and OA patients**. Expression of IL-22Rα1 in FLS isolated from PsA (*n *= 5), RA (*n *= 5) and OA (*n *= 5) patients was assessed. **(A) **A representative western blot showing expression of IL-22Rα1 in PsA, RA and OA patients. **(B) **A bar diagram showing relative intensity (R.I) of IL-22Rα1 expression in FLS obtained from PsA, RA and OA patients. Results were expressed as Mean ± SEM. Non parametric test, Kruskal Wallis with Dunn's multiple comparison test was done to determine the statistical significance.

IL-22Rα1 expression at protein level was done by western blotting. Expressions of IL-22Rα1 were found in FLS of PsA, RA and OA patients (Figure 3A). The relative intensity (R.I) of IL-22Rα1 in FLS of PsA, RA and OA patients was 0.92 ± 0.07, 0.94 ± 0.16 and 0.69 ± 0.18 respectively. There was no significant difference (Kruskal Wallis ANOVA with Dunn's multiple comparison test) in expressions of IL-22Rα1 between FLS of PsA, RA and OA patients (Figure 3B).

### Inhibitory effect of anti IL-22R antibody on rIL-22 induced FLS proliferation

**Figure 4:**
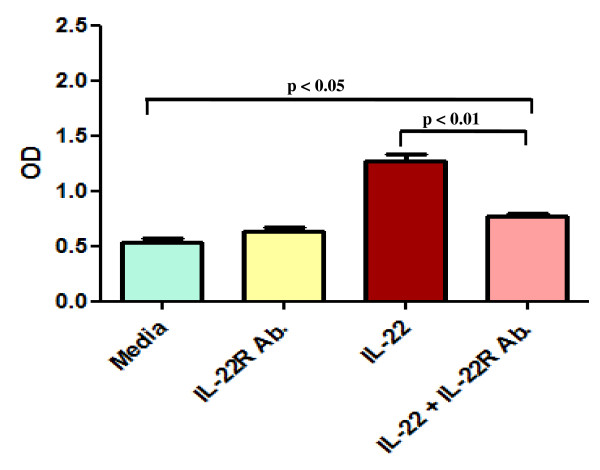
**Human anti IL-22R antibody significantly inhibited rIL-22 induced FLS proliferation in PsA**. FLS were pretreated with anti IL-22R antibody (1000 ng/ml) for 1 hr then incubated with rIL-22 (100 ng/ml) at 37°C for 5 days. MTT assay was done to evaluate the inhibitory effect of anti IL-22R antibody (IL-22R Ab). Each experiment was done in triplicate. Results were expressed as Mean ± SEM of OD. Non parametric test, Friedman's ANOVA with Dunn's multiple comparison test was done to determine statistical significance.

In MTT assay, there was a significant difference of proliferation between media (OD: 0.53 ± 0.02) and IL-22 + IL-22R Ab (OD: 0.76 ± 0.03, p < 0.05) treated FLS (Figure 4). Anti IL-22R (IL-22R Ab) antibody (OD: 0.76 ± 0.03) significantly inhibited the rIL-22 induced FLS proliferation in PsA (1.27 ± 0.06, p < 0.01, Friedman's ANOVA with Dunn's multiple comparison test) (Figure 4). There was no significant difference of proliferation between media (OD: 0.53 ± 0.02) and only IL-22R Ab (OD: 0.63 ± 0.03) treated FLS.

In FLS of RA also, IL-22R Ab (OD: 0.71 ± 0.03) significantly inhibited rIL-22 induced FLS proliferation (OD: 1.58 ± 0.05, p < 0.001, Friedman's ANOVA with Dunn's multiple comparison test. There was no significant difference of proliferation between media (OD: 0.65 ± 0.04) and only IL-22R Ab. (OD: 0.71 ± 0.03) treated FLS. In OA FLS, IL-22R Ab (OD: 0.49 ± 0.02) inhibited the rIL-22 induced proliferation (OD: 0.73 ± 0.12, p = ns), but there was no significant difference.

### Synovial fluid of PsA and RA patients are enriched with IL-22 producing CD3^+ ^T cells

CD3^+ ^T cells were obtained from synovial fluid and peripheral blood of PsA, RA and OA by magnetic bead sorting. The purity of enriched T cells was > 90% by flow cytometric analysis. Activated synovial T cells of PsA and RA produced significantly more IL-22 than that of OA. Moreover in PsA and RA, activated synovial T cells produced significantly more IL-22 than peripheral blood T cells of same patients [Table 1].

**Table 1:** IL-22 production by activated synovial fluid (SF) and peripheral blood (PB) CD3^+ ^T cells.

		IL-22 concentration (pg/ml)				
		24 hr. Incubation			48 hr. Incubation	
		Unstimulated (Mean ± SEM)	CD3/CD28 Stimulated (Mean ± SEM)	PMA/Ionomycin Stimulated (Mean ± SEM)	Unstimulated (Mean ± SEM)	PHA Stimulated (Mean ± SEM)
**PsA** **(*n *= 10)**	SF-T cells	3.85 ± 0.51	14.57 ± 0.80*^$^	18.92 ± 0.89**^#$^	4.21 ± 0.5	95.55 ± 7.81***^#$^
	PB-T cells	3.65 ± 0.23	10.44 ± 0.66*	12.83 ± 0.79*	4.28 ± 0.44	48.13 ± 6.38***^$^
**RA** **(*n *= 10)**	SF-T cells	4.22 ± 0.31	14.32 ± 0.96*^$^	18.91 ± 1.97**^#$^	4.63 ± 0.37	81.63 ± 5.90***^#$^
	PB-T cells	4 ± 0.16	10.56 ± 0.48*	11.88 ± 0.18*	4.1 ± 0.26	53.08 ± 4.58***^$^
**OA** **(*n *= 8)**	SF-T cells	4.63 ± 0.57	8.61 ± 0.79	13.59 ± 0.81*	4.79 ± 0.12	39.06 ± 3.92***
	PB-T cells	3.94 ± 0.22	6.88 ± 0.34	11.44 ± 0.78*	4.04 ± 0.18	32.78 ± 2.56***

* p < 0.05, **p < 0.01, compared to 24 hr unstimulated media; ***p < 0.001, compared to 48 hr unstimulated media, Wilcoxon matched pairs signed rank test. ^#^p < 0.05, compared to PB-T cells; ^$^p < 0.05, ^$$^p < 0.01, compared to that of OA, Mann Whitney test.

## Discussion

Several reports suggest that IL-22, a relatively new Th17 cytokine plays a critical role in the inflammation and proliferation cascade of various autoimmune diseases like rheumatoid arthritis and psoriasis [16-20,35,36]. In psoriasis, a correlation between serum IL-22 level and psoriasis area and severity index (PASI) score has been observed [28]. Higher levels of IL-22 were found in synovial fluid of PsA and RA patients [30,37]. Moreover systemic as well as intra-articular anti-TNF therapy showed reduction of increased IL-22 level in serum and synovial fluid of psoriasis and PsA patients respectively [30,38]. To our knowledge, there is no report available on the role of IL-22 in PsA. In this study we demonstrated the functional significance of IL-22 in PsA.

We observed significantly elevated levels of IL-22 in SF of PsA (17.75 ± 3.46 pg/ml) compared to OA (5.03 ± 0.39 pg/ml). We found highest concentration of IL-22 in SF of RA (21.06 ± 3.55 pg/ml), but it was not significantly different from PsA (17.75 ± 3.46 pg/ml) (Figure 1).

Most of the reports demonstrated T cells as a major source of IL-22 [18,21,22]. In addition NK cells have been reported to produce IL-22 [25] and there is one report where IL-22 was identified in FLS of RA synovial tissues [20]. Here we showed that activated synovial CD3^+ ^T cells of PsA and RA produced more IL-22 than those of OA [Table 1]. Synovial fluid from healthy volunteers could not be obtained. We chose osteoarthritis patients (OA), a non-inflammatory arthritis as a negative control. Moreover we observed that activated synovial T cells produced significantly more IL-22 compared to activated peripheral blood T cells of same patients [Table 1].

In RA, it had been shown that rIL-22 induced marked proliferation of FLS and IL-22 receptors (IL-22R1) were present in those FLS [20]. In this study, we demonstrated that human rIL-22 induced significant proliferation of FLS of PsA and IL-22Rα1 were expressed in PsA FLS (Figure 2A, B and 3A). We also confirmed the expression of IL-22Rα1 in FLS of RA and OA [20]. There was no significant difference in IL-22Rα1 expressions among PsA, RA and OA FLS (Figure 3B). We observed more proliferation of FLS with rTNF-α in comparison to rIL-22, however there was no significant difference (Figure 2A and 2B). We also showed that the rIL-22 induced proliferation of FLS is inhibited by IL-22R antibody in PsA (Figure 4). These observations suggest that functionally active IL-22 is present in increased amount in the SF of PsA.

We observed a novel finding in PsA and RA that combination of rTNF-α and rIL-22 induced significantly more proliferation of FLS than rIL-22 alone (Figure 2A and 2B). In few studies on human keratinocytes, it have been shown that IL-22 together with TNF-α produce synergistic effect in terms of secretion of antimicrobial peptides and chemokines [39,40] however to our knowledge there is no study showing the combined effect of IL-22 and TNF-α on the proliferation of FLS. In this study we did not elucidate the mechanism responsible for this combined effect of IL-22 with TNF-α in FLS proliferation. Both TNF-α and IL-22 exert proliferative effect by inducing phosphorylation of extracellular signal regulated kinases (ERK)/p38 MAPK [20,41]. In a study on keratinocytes, Eyerich *et al *showed the synergistic effect of IL-22 and TNF-α on induction of immune genes by phosphorylation of different MAPK specially p38 [39]. Thus it is possible that combination of IL-22 and TNF-α induce more phosphorylation of Erk/MAPK pathway than either of IL-22 or TNF-α alone resulting in more proliferation of FLS.

## Conclusions

To summarize, in this study we observed significantly elevated levels of IL-22 in synovial fluid of PsA compared to OA patients and activated synovial T cells produced more IL-22 than activated peripheral blood T cells in PsA. IL-22 is functionally active since it induced significant proliferation of FLS. Thus IL-22 has the potential to contribute in the pannus formation. Moreover, IL-22 in combination with TNF-α induced significantly more proliferation of FLS than IL-22 or TNF-α alone which provides a new insight in the pathogenesis of PsA. Here we have also shown that the IL-22 induced FLS proliferation can be blocked significantly by anti IL-22R antibody. Thus, IL-22/IL-22R system may be a potential novel target of drug development for psoriatic arthritis.

## Abbreviations

CFSE: carboxy fluorescein succinimidyl ester; ELISA: enzyme-linked immunosorbent assay; FLS: fibroblast like synoviocytes; MTT: [3-(4,5-Dimethylthiazol-2-yl)-2,5-Diphenyltetrazolium Bromide]; ns: not significant; OA: osteoarthritis; OD: optical density; PB: peripheral blood; PBMC: peripheral blood mononuclear cells; PHA: phytohaemagglutinin; PMA: phorbol myristate acetate; PsA: psoriatic arthritis; RA: rheumatoid arthritis; rIL-22: recombinant interleukin 22; rTNF-α: recombinant tumor necrosis factor alpha; SF: synovial fluid; SFMC: synovial fluid mononuclear cells.

## Competing interests

The authors declare that they have no competing interests.

## Authors' contributions

AM performed all experiments with help from others and did the statistical analysis. SKR designed experiments, supervised statistical analysis. SPR conceived the study and helped in designing experiments. All authors have contributed to the conception and/or acquisition of data and analysis for this project and to either drafting or revising the manuscript. All authors read and approved the final manuscript.
